# A New Aerosol‐Based Photobioreactor for the Cultivation of Cyanobacteria on Luffa

**DOI:** 10.1002/bit.28992

**Published:** 2025-04-13

**Authors:** Jonas Kollmen, Andreas Stiefelmaier, Ramtin Mofrad, Dorina Strieth

**Affiliations:** ^1^ RPTU Kaiserslautern‐Landau Institute of Bioprocess Engineering Kaiserslautern Germany; ^2^ Math2Market Kaiserslautern Germany

**Keywords:** attached cultivation, biofilm, cyanobacteria, EPS, photobioreactor

## Abstract

Cyanobacteria are promising organisms for sustainable biotechnology due to their ability to grow photoautotrophically and their wide range of products. Many cyanobacteria grow in the form of biofilms, which is why the development of photobioreactors (PBR) for the cultivation of cyanobacteria in the form of biofilms is of great interest. However, these biofilm PBR are mostly based on artificial growth surfaces, whereas biodegradable growth surfaces would be favored in terms of sustainable production and application. Luffa sponges (the dried fruit of *Luffa cylindrica*) are excellent biodegradable growth surfaces for cyanobacteria. Therefore, a biofilm PBR for cultivation of cyanobacteria on Luffa was developed in this study. Since many cyanobacteria grow naturally as biofilms in an air‐exposed form and this should be imitated to improve growth, an aerosol‐based PBR (abPBR) should be used for cultivation. This involves supplying the cyanobacteria with a nutrient mist. The abPBR was comprehensively characterized by determining the distribution of light, humidity and temperature inside the reactor. In addition, the residence time distribution of the aerosol was determined both experimentally and simulatively. In final cultivation experiments, it was shown that the abPBR is ideal for cultivating cyanobacteria and at the same time the aerosol system enables a simple imitation of drought stress. With the cyanobacteria *Nostoc* spec. and *Desmonostoc muscorum*, maximum area‐time‐yields (ATY) in relation to the growth surface of 6.34 and 4.19 g m^−2^ d^−1^, respectively, were achieved. Compared to previously developed abPBR, the ATY has been increased by a factor of 2.3.

## 1 Introduction

Cyanobacteria, phototrophic prokaryotes, are interesting production organisms, due to their ability to grow photoautotrophically, which enables sustainable cultivation without additional carbon sources. However, special reactor systems are required to cultivate cyanobacteria, as a sufficient supply of light energy is essential. A basic distinction is made between two cultivation systems—open and closed. Open systems are relatively simple setups that do not require a high level of technical equipment. These can be open ponds or raceway ponds, which have a directed flow. The common feature of open cultivation systems is that their construction is relatively simple and therefore affordable. Upscaling open or raceway ponds is also relatively simple. The disadvantages, however, are their high space requirement (upscaling is only possible through horizontal expansion) and some disadvantages of the open construction itself, such as an increased risk of contamination. Completely open cultivation is only possible in climatically stable regions. In regions with high rainfall or large temperature fluctuations, placing the open ponds indoors is an alternative (Roselet et al. 2013).

Photobioreactors (PBR) are closed systems that have been and continue to be established in many different designs. The most common designs are tubular, vertical column and flat panel PBRs (Ahmad et al. 2021). In general, PBRs have the advantage that the cultivation conditions are more controllable than in open systems. Due to the closed design, they are also less susceptible to external influences and contamination. In contrast to open systems, the space requirement can be reduced by extending the system vertically. Compared to open systems, the higher costs and the greater complexity in terms of design and operation are disadvantageous. Another problem is the upscaling of PBR, especially if a constant light input is to be guaranteed, as this prevents up‐scaling across the diameter of the systems. Nevertheless, PBRs offer a much better platform and greater potential for optimizing the cultivation of cyanobacteria. The various PBR designs have already been described in detail in various reviews (Ahmad et al. 2021; Chang et al. 2017; Gupta et al. 2015; Singh and Sharma 2012). Biofilm PBRs are a special form of PBRs that has gained attention in recent years. Biofilm PBRs are based on the principle that some cyanobacteria show better growth in biofilms than in suspension, as this reflects their natural growth behavior. For this purpose, in biofilm PBRs, the cyanobacteria are provided with a growth surface on which the biofilm can develop. The supply of media can then take place, for example, by repeated immersion in medium or constant overflow with medium. By using carriers as growth support, submerged systems can also be expanded to biofilm PBR. Table 1 gives an overview of existing biofilm PBRs. Benefits of biofilm PBR are reduced costs due to increased productivity and improved water to biomass ratio. To avoid the disadvantages, as limited light supply, of high biofilm heights, regular harvesting is necessary. Aerosol‐based PBRs (abPBRs) are a special form of biofilm PBR. Here, the biofilm is supplied with medium in the form of an aerosol, which is generated using ultrasonic atomizers, for example, and directed over the biofilm. The medium can be recycled through a closed cycle, thus saving resources. The aerosol‐based system is particularly advantageous for cultivation of terrestrial biofilm‐forming cyanobacteria, as their natural growth conditions are simulated. Stress situations, such as dry phases, can also be easily realized with an abPBR. Several generations of abPBRs for the cultivation of cyanobacteria have been developed in recent years. The first generation, called ePBR (Emersed PhotoBioReactor), was developed by Kuhne et al. (2014) and subsequently further developed and optimized by Strieth et al. (2021). By using ultrasonic atomizers, the aerosol can be produced either directly in the reactor or outside in separate vessels. In the latter case, the aerosol must be transported from the atomizer vessel to the reactor, for example, by compressed air. In these ePBRs, sticks made of plastic, silicone or glass were used as growth surfaces, some of which can also be illuminated from the inside. Other geometries in the form of a hexagonal ePBR were also developed (Stiefelmaier 2020). In the hexagonal ePBR, the illumination is placed outside the reactor and is possible with different wavelengths. abPBRs also offer potential for use as facade reactors, with an appropriately adapted geometry (Scherer et al. 2020; Schmidt et al. 2017). This allows areas that cannot be used for agriculture to be utilized for the cultivation of cyanobacteria and microalgae. Aerosol‐based bioreactors have not only been developed for the cultivation of phototrophic organisms, but also for the heterotrophic cultivation of pro‐ and eukaryotic organisms (Hornung et al. 2007; Tscheschke et al. 2015; Weathers et al. 2008).

**Table 1:** Comparison of biofilm photobioreactors for the cultivation of cyanobacteria and microalgae on substrates.

Reactor	Substrate	Biofilm placement	Strain	ATY_cultivation surface_ (g_CDW_ m^−2^ d^−1^)	ATY_Footprint_ (g_CDW_ m^−2^ d^−1^)	STY (g_CDW _L^−1^ d^−1^)	Reference
abPBR	Luffa	Air‐exposed	*Nostoc* sp.	6.34	7.31	0.114	This study
STR	HDPE	Submerged	*Nostoc* sp.	0.11	0.13	0.103	Walther et al. (2022)
psMBPBR	HDPE	Submerged	*Nostoc* sp.	0.96	4.19	0.018	Walther et al. (2022)
lsMBPBR	HDPE	Submerged	*Nostoc* sp.	1.84	3.25	0.026	Walther et al. (2022)
ePBR	glass	Air‐exposed	*Nostoc* sp.	2.68	4.64	0.134	Strieth (2019) and Strieth et al. (2021)
ePBR	PMMA	Air‐exposed	*Nostoc* sp.	2.42	4.78	0.121	Strieth (2019) and Strieth et al. (2021)
ePBR	Silicone	Air‐exposed	*Nostoc* sp.	2.78	4.32	0.139	Strieth (2019) and Strieth et al. (2021)
Attached algal culture system	Polystyrene foam	Partly submerged	*Chlorella* sp.	2.57	2.57	0.175	Johnson and Wen (2010)
Suspended‐solid phase PBR	Cotton, mohair	Submerged	*Scenedesmus* LX1	—	4.58	0.021	Lin‐Lan et al. (2018)
Algal biofilm airlift PBR	Fiber	Submerged	*Chlorella vulgaris*	0.82	3.19	0.016	Tao et al. (2017)
Biofilm membrane PBR	Fiber	Submerged	*Chlorella vulgaris*	0.44	5.41	0.022	Peng et al. (2020)
Revolving algal biofilm	Cotton duct	Partly submerged	*Chlorella vulgaris*	5.1	18.92	—	Gross and Wen (2014)
Parallel plate microalgae biofilm reactor	Polycarbonate sheet	Submerged	*Scenedesmus obliquus*	2.5	2.5	0.26	Zamalloa et al. (2013)
Algae biofilm PBR	Concrete	Partly submerged	*Botryococcus braunii*	0.71	0.71	0.019	Ozkan et al. (2012)
Rotating algal biofilm reactor – lab scale	Cotton rope	Partly submerged	mixed biofilm	—	5.5	—	Christenson and Sims (2012)
Rotating algal biofilm reactor – pilot scale	Cotton rope	Partly submerged	mixed biofilm	—	31	—	Christenson and Sims (2012)
Falt plate parallel horizontal PBR	Glass	Submerged	*Nitzschia palea*	2.8	—	—	Schnurr et al. (2013)
Parallel plate airlift PBR	Cellulose acetate	Submerged	Mixed biofilm	2.08	—	0.0482	Genin et al. (2014)
Horizontal flow lanes	Polyfelt sheet	Submerged	Mixed biofilm	9.9	—	—	Boelee et al. (2014)
Multi‐layer PBR	Glass	Air‐exposed	*Botryococcus braunii*	0.091	0.41	—	Cheng et al. (2013)
Multi‐skin sheet emerse PBR	Polycarbonate	Air‐exposed	*Coleofasciculus chthonoplastes*	1.7	9.47	—	Scherer et al. (2020)
Capillary driven PBR	Polyester microfibers	Air‐exposed	*Scenedesmus* sp.	0.375	9.6	—	X.‐Q. Xu et al. (2017)
Twin‐layer PBR	Paper	Air‐exposed	*Phaeodactylum tricornutum*	0.886	—	—	Naumann et al. (2013)
Biofilm cultivation system	Cellulose acetate/nitrate membrane	Air‐exposed	*Haematococcus pluvialis*	6.0	0.11	0.2	Yin et al. (2015)
Attached biofilm reactor	Filtration membrane	Air‐exposed	*Pseudochlorococcum* sp.	8.0	0.125	—	Ji et al. (2014)
Attached PBR	Filter paper	Air‐exposed	*Scenedesmus* sp.	6.2	—	—	Cheng et al. (2020)
Fixed‐bed biofilm reactor	Porous substratum canvas	Air‐exposed	*Chlamydomonas* sp.	3.106	49.70	—	Shen et al. (2019)
Rotating biological contactor	Stainless steel woven mesh	Partly submerged	*Chlorella sorokiniana*	20.1	10.64	0.043	Blanken et al. (2014)
Photorotating biological contactor	PVC	Partly submerged	*Klebsormisium* sp.	0.447	5.279	0.048	Orandi et al. (2012)
Rotating flat plate PBR	PVC	Partly submerged	*Chlorella vulgaris*	0.42	2.99	—	Melo et al. (2018)
Rotating algal biofilm reactor	Cotton	Partly submerged	Mixed biofilm	—	0.96	—	Iman Shayan et al. (2016)
Attached cultivation	Nylon	Submerged	Mixed biofilm	5.66	9.10	0.023	Lee et al. (2014)
Algal turf scrubber	Polyethylene	Submerged	Mixed biofilm	5.00	5.00	0.025	Mulbry and Wilkie (2001)
Horizontal flat panel PBR	Concrete	Submerged	Mixed biofilm	12.21	—	0.244	Sukačová et al. (2015)
Algal biofilm reactor	Nonwoven spun bond fabric	Submerged	Mixed biofilm	4.0	—	0.084	Choudhary et al. (2017)
Attached PBR	Cotton	Submerged	Mixed biofilm	1.91	—	—	de Assis et al. (2019)
Raceway ponds	—	—	—	—	6.2–24.5	0.03–0.19	de Vree et al. (2015)
Flat panel PBR	—	—	—	—	8–27.5	0.15–1.2	de Vree et al. (2015)
Horizontal tubular PBR	—	—	—	—	5.8–19.5	0.12–0.85	de Vree et al. (2015)
Vertical tubular PBR	—	—	—	—	5.8–24.4	0.31–1.45	de Vree et al. (2015)

Abbreviationss: abPBR, aerosol‐based PBR; ATY, area time yield; ePBR, emerse PBR; lsMBPBR, lab‐scale moving bed PBR; PBR, photobioreactor; psMBPRB, pilot‐scale MBPBR; STR, stirred tank reactor; STY, space time yield.

Biofilm PBRs are developed for the cultivation of phototrophic biofilms. Phototrophic biofilms are biofilms that contain photosynthetically active organisms such as microalgae or cyanobacteria, whereby the focus in this study lies on cyanobacteria. Cyanobacteria are prokaryotes, which are among the oldest organisms on Earth (Hedges et al. 2001). They are able to grow photoautotrophically as well as heterotrophically (also mixotrophically), which is one reason for their high adaptability (Schwarz et al. 2020). As a result, cyanobacteria can be found in almost all regions of the world. Just like higher plants, they are capable of photosynthesis and contain chlorophyll and carotenoids as photopigments. Furthermore, cyanobacteria possess additional accessory pigments, the phycobiliproteins, which allow them to use a wider spectrum of light (Li et al. 2019). Cyanobacteria grow embedded in a matrix of extracellular polymeric substances (EPS). The EPS consists mainly of water, polysaccharides, proteins, and lipids (H. Xu et al. 2013). They play an essential role in biofilm formation by allowing cells to attach to each other and to surfaces (Rossi and De Philippis 2015). They also serve as a water reservoir in the biofilm. The high water retention capacity of cyanobacterial biofilms is one reason why they are gaining interest for use as biofertilizers (Kollmen and Strieth 2022). In this context, biofilms are beneficial as they can reduce soil erosion and increase soil moisture. In addition, cyanobacteria can act as nitrogen suppliers to crops. Some species are able to fix N_2_ from the atmosphere and release it in a form that plants can use, such as ammonium or amino acids (Stal 2005). The beneficial effects of cyanobacteria on plant growth have been widely studied and the interested reader is referred to further literature (Kollmen and Strieth 2022). Co‐cultures between cyanobacteria and plants have already been successfully carried out and by cultivating cyanobacteria as a biofilm, they can be directly adapted for later use as a biofertilizer. However, this raises the question of how the biofilm can be removed from the carriers or the growth surface. A simple solution would be to spread the biofilm adherently on the carriers. However, most industrially used carriers are made of plastic, which is why they are unsuitable for use in agriculture (release of plastic into the environment). Degradable carriers are a biologically acceptable alternative, and Luffa has proven to be a particularly suitable carrier for the cultivation of cyanobacteria (Kollmen et al. 2023). Luffa is the dried fruit of the plant *Luffa cylindrica*, which after drying consists of a network of fine fibers with a high porosity and surface area (Ogbonna et al. 1994). The fact that Luffa is biodegradable makes it possible to cultivate cyanobacteria on the Luffa and subsequently apply the carriers including biofilm as biological fertilizer. It has already been shown that microalgae and/or cyanobacteria immobilized on Luffa can be used to remove heavy metal ions in wastewater treatment or to capture carbon dioxide (Akhtar et al. 2003a, 2004; Akhtar et al. 2008; Akhtar et al. 2003b; In‐na et al. 2020). In the latter application, the cyanobacteria were immobilized in a latex layer on the Luffa to produce a biocomposite. In‐na et al. (2022) also performed a techno‐economic analysis of CO_2_ fixation with a cyanobacteria Luffa biocomposite and showed that the biocomposite leads to a significant reduction in water and energy demand. Thus, initial approaches to the technical use of cyanobacteria immobilized on Luffa already exist, but the cultivation of cyanobacteria on Luffa has not yet been intensified. So, this study presents a key innovation that combines the advantages of Luffa and abPBR to optimize the immobilized cultivation of cyanobacteria on Luffa.

Therefore, a reactor system is developed and established in this study, which enables the cultivation of cyanobacteria on commercially available Luffa. The medium is supplied in the form of an aerosol due to the advantages of abPBR described above. The possibilities of adapting the biofilm by specifically changing the aerosol supply are also being investigated. The residence time of the medium in the reactor is determined both experimentally and simulatively. Furthermore, the distribution of light, temperature, and humidity in the reactor are investigated.

## 2 Material and Methods

### 2.1 Development and Setup of the abPBR

**Figure 1:**
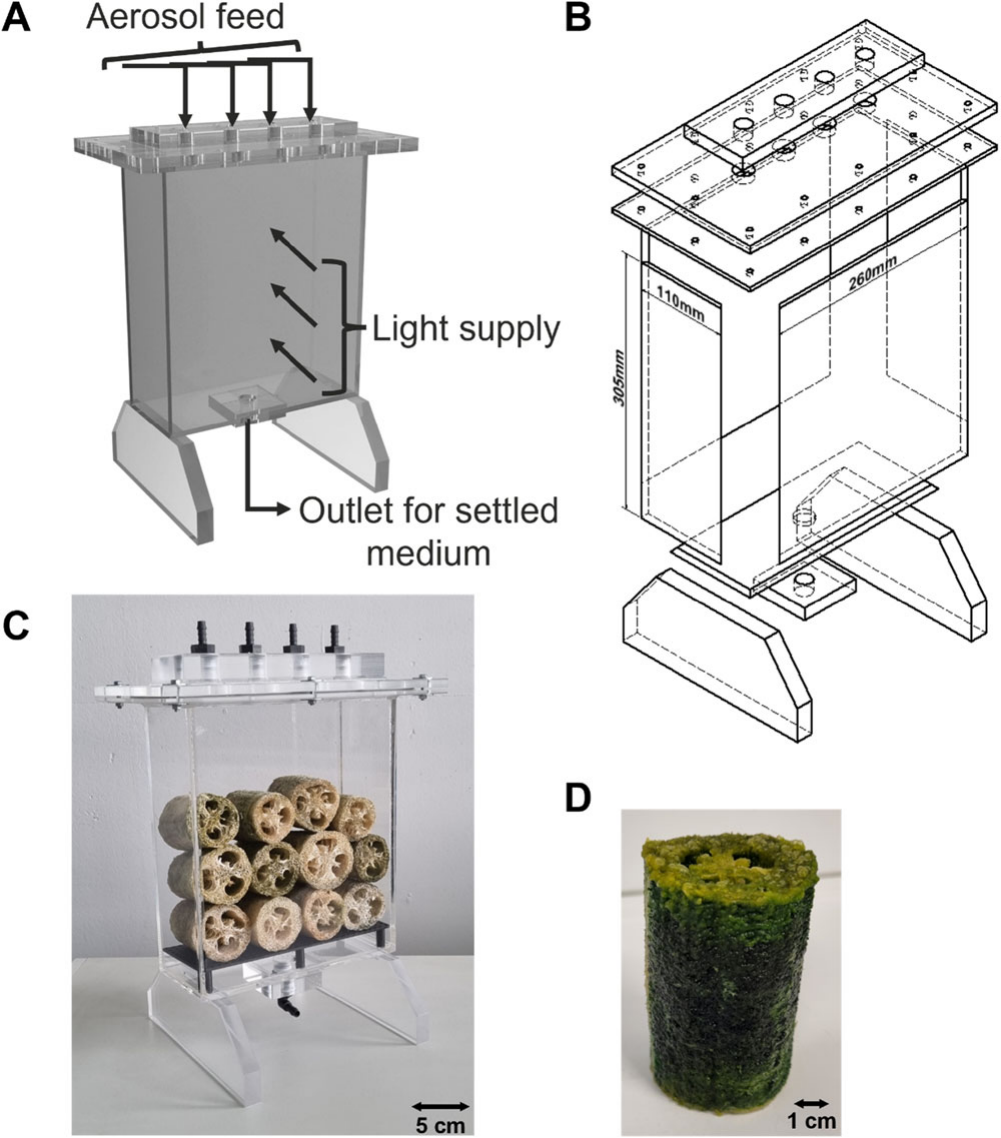
Design of the aerosol‐based PBR (abPBR) for the cultivation of cyanobacteria on Luffa. (A) 3D rendering of the abPBR. (B) Exploded drawing of the components of the abPBR including external dimensions of the cultivation chamber. (C) Picture of the abPBR filled with Luffa. (D) Luffa covered with biofilm of *Desmonostoc muscorum* after cultivation in the abPBR.

Polymethyl methacrylate (PMMA) was chosen as the material for the construction of the reactor as it combines high‐light transmission with low weight. All components consist of 10 mm thick PMMA panels. Only the components with threads (inlets and outlets) were made thicker to increase stability and prevent the PMMA from tearing when connectors are connected several times. All components were glued with Acrifix (1 R 0192, Röhm GmbH, Weiterstadt, Germany). The geometry of the reactor is based on a plate reactor with inner dimensions of 25 × 10 x 30 cm (L x D x H, see Figure 1). The depth of 10 cm allows the use of commercially available Luffa, which are usually sold with a length of 10 cm. Moreover, shallower depths are a constraint when assembling the reactor and make cleaning more difficult. The influence of reactor depth on light loss was analyzed separately (see Section 3.2). The reactor is filled from above. The lid can be removed from the reactor for this purpose. For sealing, an ethylene propylene diene monomer rubber flat seal (Fugendichtband24 GmbH, Freudenberg, Germany) is installed over the entire surface between the lid and reactor. The connection is made using 12 M6 screws, which allow the reactor to close evenly. There are four openings in the lid, each with a ½" thread. These allow hose nozzles with an internal diameter of 8 mm to be screwed in, which are used to supply the reactor with aerosol. Each connection is located in the center above a Luffa. An outlet is provided at the bottom of the reactor, also with a ½" thread for screwing in a hose nozzle, and a 3D printed perforated plate made of polypropylene was used to prevent the bottom row of Luffa from lying on the floor. This has holes with a diameter of 5 mm to allow the aerosol or medium to pass through. The aerosol was supplied in the same way as the ePBR 2 from Strieth et al. (2021) via external nebulization combined with recycling of the medium. The aerosol was produced with an ultrasonic nebulizer (NB‐80E‐01, TDK Europe, München, Germany) and transported through the system with compressed air at a flow rate of 1 L min^−1^. Due to the material selected, the reactor was sterilized with a 5% sodium hypochlorite solution for all experiments.

Cultivation in the abPBR was carried out in a heating chamber (IPS 749, Memmert, Germany) for temperature control. Lighting was provided by a total of 8 (four per side) LED strips with an output of 30 W each. The photosynthetically active radiation (PAR) at the reactor surface with these lamps equals 250 µmol m^−2^ s^−1^. Two abPBRs could be operated in parallel. The aerosol was generated with an atomizer unit using ultrasound (Strieth et al. 2021).

### 2.2 Light, Humidity, and Temperature Distribution

Temperature and humidity distribution were measured during the cultivation using iButton (DS1923, Maxim Integrated Products, San José, California, USA) sensors. The sensors were placed on the inner wall of the reactor at various heights (lower and upper third) and inside the Luffa. Temperature and humidity were measured continuously every 10 min. Light distribution was measured using a quantum sensor (MQ‐500, Apogee Instruments, Logan, Utah, USA) at defined distances from the light source. The light loss at different depths of the Luffa was also determined.

### 2.3 Determination of the Aerosol Residence Time in the abPBR

The mean dynamic residence time of the aerosol in the abPBR was determined by measurement of the relative humidity in different areas of the reactor. For this purpose, the reactor was first filled with Luffa to imitate the cultivation conditions in the system. Deionized water was then transferred from the storage vessel to the nebulizer unit and atomized into aerosol spray. The aerosol was fed into the reactor using compressed air at a flow rate of 1 L min^−1^, while the humidity was measured continuously. iButton sensors (DS1923, Maxim Integrated Products, San José, California, USA) were used to measure the relative humidity. The sensors were placed at the inlet and at the outlet of the reactor. The humidity at the start of the measurement (dry reactor) was subtracted from all other values, as this value corresponds to a reactor without aerosol. The values were then normalized and divided by the reactor volume of 7.5 L, to calculate the aerosol concentration in the reactor. This was done under the assumption that the normalized humidity can only assume values between one and zero, since *c*(*t*) < *n*_0_, where *n*_0_ = relative humidity at time *t*_0_, applies at all times. The test time *t* is replaced by the dimensionless time *θ*_*t*_.

### 2.4 Simulation of the Aerosol Distribution in the abPBR

The aerosol distribution in the reactor was investigated using computional fluid dynamics (CFD) using the GeoDict software (developed by Math2Market GmbH, Kaiserslautern, Germany).

### 2.5 Pre‐Culture

The strain *Desmonostoc muscorum* 90.3 from the strain collection of Prof. Dr. Burkhard Büdel (former Department of Biology, Chair for Plant Ecology and Systematics, RPTU in Kaiserslautern) was used for cultivation in the abPBR. The pre‐culture was carried out in 300 mL Erlenmeyer flasks without baffles using 100 mL BG11 medium according to Stanier et al. (1979). The incubation was carried out at 120 rpm and 30°C in an incubator (Multitron S 000115689, Infors HT, Bottmingen, Switzerland). During cultivation, the pre‐cultures were continuously illuminated with a light intensity of 120 μmol_photons_ m^−2^ s^−1^.

### 2.6 Cultivation in the abPBR

Before cultivation, the Luffas were dried for 24 h in a drying chamber at 60°C and their dry weight was determined the next day after cooling to room temperature. The biomass was harvested from the pre‐culture by centrifugation at 8000 x g for 15 min and the supernatant was discarded. To inoculate the Luffas, 0.5 g of biomass was suspended in 10 mL of BG11 medium and evenly distributed to one Luffa each using a 5 mL pipette. The inoculated Luffas were then placed in the abPBR. One row contained four Luffa, and the reactors were filled with two rows. The Luffa were not fixed in the reactor and were not connected to each other to enable partial harvesting. As the dimensions of the reactor are adapted to the Luffa, there is no risk of displacement during cultivation. The aerosol was supplied continuously or at intervals, depending on the experiment. The intervals can be adjusted depending on the desired influence on cultivation, for example, to imitate drought stress. In this study, intervals of 4 h on and 4 h off and 12 h on followed by 12 h off were used. The aerosol was transported using a compressed air flow of 1 L min^−1^. The medium was recycled via a floor drain. The illumination was carried out in a rhythm of 16 h on and 8 h off with a PAR of 250 µmol m^−2^ s^−1^ at the outer surface of the abPBR. Cultivation was carried out for 14 days. At the end of cultivation, the entire Luffas including biomass were removed from the reactor and subjected to further analysis.

### 2.7 Biomass Determination and EPS Extraction

The biomass was harvested from the Luffa by multiple rinsing with deionized water followed by EPS extraction according to Kollmen et al. (2025). The biomass and EPS were lyophilized at 1 mbar and −20°C for 24 and 48 h, respectively. Cell dry weight of the rinsed biomass (CDW_rinsed_) and EPS were then determined gravimetrically. To be able to determine the biomass remaining on the Luffa, these were then dried at 60°C until their weight was constant and weighed. The weight (m_Luffa, end_) was offset against the initial weight of the Luffa (*m*_Luffa,0_) before cultivation to determine the remaining CDW. The CDW is always given as the sum of the dry biomass remaining on the Luffa and the dry biomass rinsed from the Luffa for analysis.

(1) $$\text{CDW}\;=\;m_{\text{Luffa},\text{end}}-\;m_{\text{Luffa},0}+{\text{CDW}}_{\text{rinsed}}$$

### 2.8 Analyses of Growth Behavior

For comparison of different PBRs three reference values were determined. The area‐time‐yield (ATY) was calculated (i) in relation to the cultivation surface (S) available for the biofilm (ATY_cultivation surface_) and (ii) in relation to the footprint (A) of the reactor (ATY_footprint_). With the cell dry weight of the inoculum (CDW_start_) and the cultivation time (*t*) the ATY was determined as:

(2) $${\text{ATY}}_{\text{cultivation surface}}=\frac{\text{CDW}\;-{\text{CDW}}_{\text{start}}}{t\;\times \;S}$$

(3) $${\text{ATY}}_{\text{footprint}}=\frac{\text{CDW}\;-{\text{CDW}}_{\text{start}}}{t\;\times \;A}$$

As a third reference value, the space‐time‐yield was calculated in relation to the cultivation volume (*V*) of the reactor was determined:

(4) $$\text{STY}\;=\;\frac{\text{CDW}\;-\;{\text{CDW}}_{\text{start}}}{t\;\times \;\text{V}}$$

Phycobiliproteins were extracted according to the method described by Kollmen et al. (2025).

### 2.9 Statistical Analysis

The experiments were carried out in biological replicates (exact number can be found in the respective caption) and mean values and standard deviations were calculated from them. The Mann–Whitney *U* test was performed to determine significant differences in the data.

## 3 Results and Discussion

### 3.1 Light, Humidity, and Temperature Distribution

The growth of cyanobacteria is essentially influenced by the parameters cultivation temperature, light availability, and the supply of nutrients. The parameters of light intensity, humidity, and temperature at various points in the reactor can provide a good indication of the success of a cultivation. The light intensity is mainly influenced by three factors: the Luffa, the aerosol, and the distance from the light source. Therefore, the light loss was measured at different distances from the light source with three setups: (i) only air between light source and sensor, (ii) aerosol between light source and sensor, and (iii) a piece of Luffa of different length between light source and sensor. During cultivation all three factors are added up.

**Figure 2:**
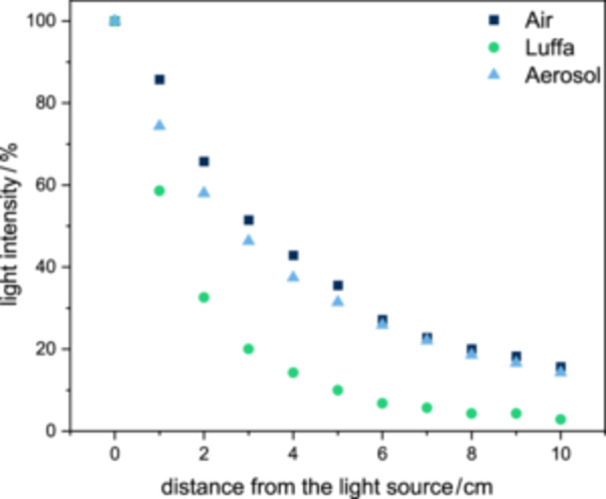
Light intensity as a function of the distance to the light source. The light intensity was measured through three materials: Air (rectangle), Luffa (circle), and aerosol (triangle).

The light intensity was mainly influenced by the distance between the light source and the measuring point (see Figure 2). For example, the loss of light intensity at 10 cm was approximately 85%. If the intermediate space is additionally filled with aerosol at a density similar to that in the reactor, the effects on the loss of light were low and only around 4% on average. The effect decreased with increasing distance from the light source. If the illumination was provided through a piece of the Luffa, the loss of light intensity was further increased compared to the aerosol. As a result, only around 3% of the original light intensity reached the sensor when illuminated through a 10 cm long piece of Luffa. However, this depended on the structure and the point at which the illumination took place. In the experiment, the light loss was determined by the inner part of the Luffa, which has a higher porosity than the outer part. Therefore, complete shadowing could occur in other areas even at lower depths. Based on the results, the reactor was illuminated from the front and rear in the experiments, resulting in greater light intensity inside the Luffa. After 5 cm of Luffa, the light loss was still 90%, but by illuminating from two sides, this was halved in the center of the Luffa and the illumination from both sides could also reach the biomass at further depths. Nevertheless, greater biomass growth is expected in the outer areas of Luffa. The loss of light through Luffa was also determined by In‐na et al. (2020). They measured the light through different parts and with different orientations of the Luffa. The most comparable measurement is the measurement through a whole Luffa in a vertical direction, in which they determined 1.79% light loss per mm of Luffa. This would mean that no more light would pass through the Luffa after approx. 5.6 cm. At the maximum illumination depth of 5 cm, which is used in this study, light would still reach the Luffa according to In‐na et al. (2020).

**Figure 3:**
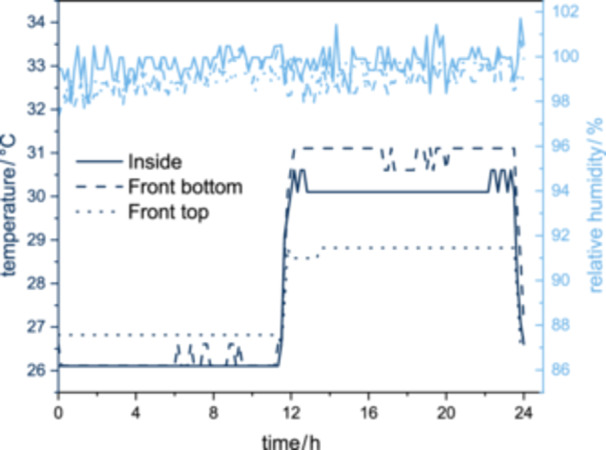
Temperature and humidity curve in the abPBR over time. The aerosol supply (1 L min^−1^) was switched off after 12 h and switched on again after 24 h. The lighting was provided from outside (see Section 2.1) and was switched off for the first 12 h and then switched on again for 12 h.

The humidity was almost constant during cultivation and was also independent of the location in the reactor (see Figure 3). In the present case, the aerosol supply was operated in a 12 h on/12 h off rhythm. As Luffa can absorb and store a large amount of water (Alhijazi et al. 2020), it can be assumed that the humidity in the reactor remains constant even when the aerosol supply is switched off due to the moisture in the sponges. This applies when the aerosol supply is switched off for at least up to 12 h. Strieth (2019) investigated the moisture distribution in an ePBR, observing a decrease in humidity in the reactor when the aerosol supply was switched off. As there was no Luffa in the reactor here, the assumption that Luffa can store water and thus keep the humidity in the reactor constant over long periods of time can be confirmed. The temperature in the reactor depends on the lighting, as the reactor heated up during the lighting phase due to radiant heat. As a result, the temperature increased by up to 5°C during the lighting phase, depending on the location in the reactor. The smallest increase was recorded in the upper area of the reactor (2°C), as the lighting was only located in the lower area of the reactor, which was filled with Luffa. The temperature difference between the surface of the reactor (the point closest to the lighting) and the center of the reactor was approximately 1°C. It can therefore be assumed that the heat was evenly distributed over the Luffa sponges. When the lighting was switched off, the temperature throughout the reactor was approximately equal to the temperature set in the incubation chamber. The increase in temperature due to the lighting must be considered during cultivation and strain selection to limit negative effects.

### 3.2 Aerosol Residence Time

**Figure 4:**
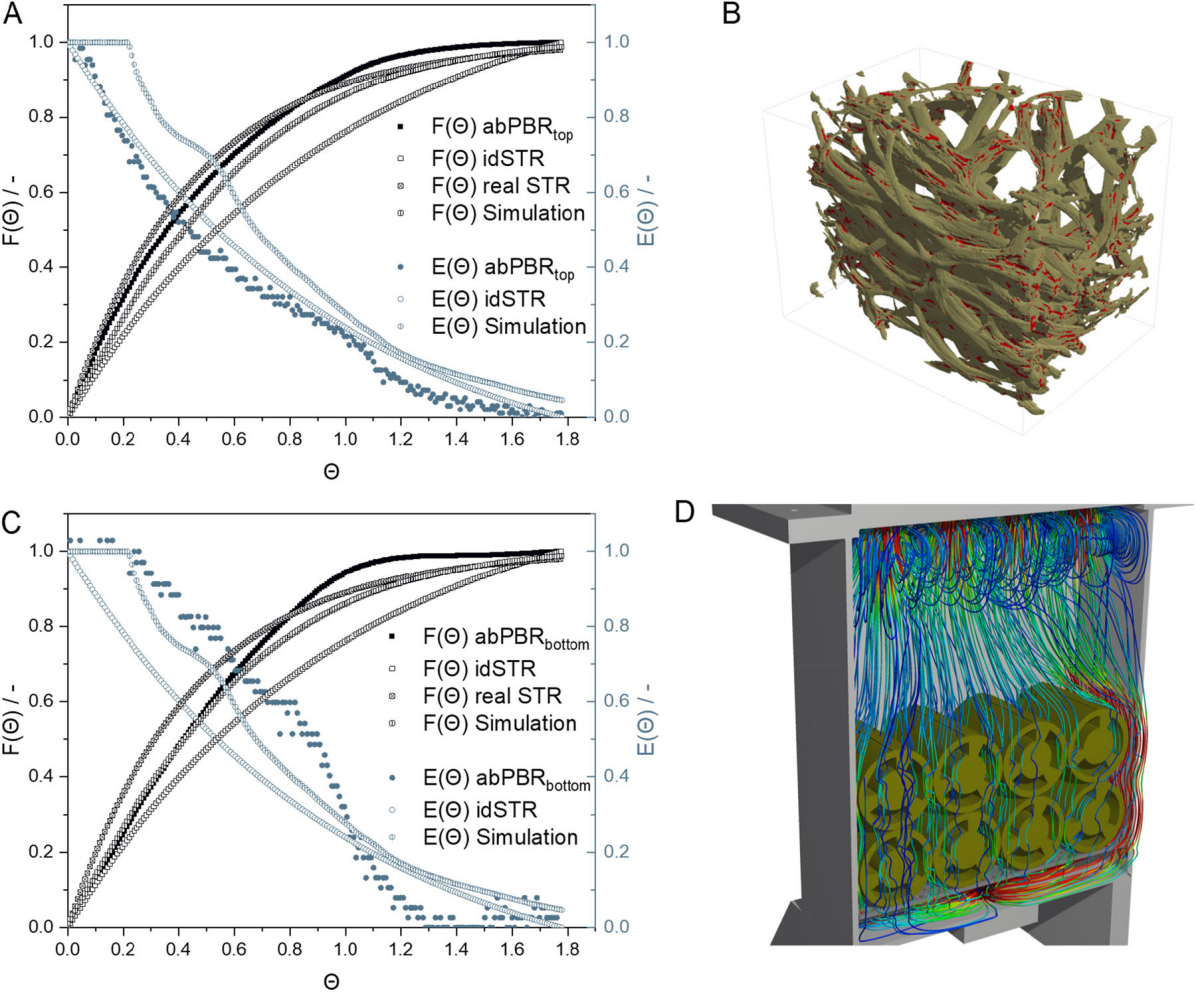
Experimental and simulative determination of the residence time distribution in abPBR. (A and C) Experimentally determined RTD and CDF in the upper third of the abPBR (abPBR_top_, A) or in the lower third of the abPBR (abPBR_bottom_, C), calculated RTD and CDF for an ideal stirred reactor (idSTR) using the volume of the abPBR, calculated CDF for an ideal STR considering the dead space volume (real STR) according to Müller‐Erlwein (2015) and simulated RTD and CDF for abPBR (Simulation). (B) µCT Scan of a piece of Luffa (1 × 1 cm side length) with filled inner pores (red) used for the calculation of physical parameters for the simulation of RTD and CDF. (D) Simulated flow lines in the abPBR with compressed air flow at 1 L min^−1^.

The residence time is generally an important parameter for characterizing bioreactors. In the abPBR, the distribution and residence time of the aerosol in the reactor play an important role, as this influences the supply of nutrients to the biofilm. Therefore, the residence time distribution (RTD) of the aerosol was experimentally and simulatively (via CFD simulation) determined. In the experimental setup, the moisture measurement, which is the tracer for determining the RTD, was carried out at two different points in the reactor, in the upper and lower third of the reactor. This was done to determine the influence of the flow and the Luffa in the reactor on the aerosol distribution. The RTD function *E*(*t*) and the cumulative distribution function (CDF) *F*(*t*) were calculated to compare the simulated with the experimentally determined data (see Figure 4). A complete mixing of the aerosol was assumed. The RTD function and the CDF were calculated based on formulae for the ideal stirred tank reactor (idSTR). This represents one of the ideal limiting cases (i) the ideal flow tube and (ii) the idSTR. The RTD calculated from the experimental values in the upper part of the reactor was similar to the RTD for an idSTR. In contrast, the RTD from the simulated data and from the experimental values from the lower part of the reactor differed significantly. Since the simulated data were determined at the outlet of the reactor, the agreement with the experimental data in the lower part is logical. Both curves showed an uneven gradient, which results from the flow through the Luffa. The fact that the aerosol flow can partly flow past the Luffa and another part flows through the Luffa at a significantly lower speed (see Figure 4D) resulted in a decrease in the speed of the concentration change after an initial rapid increase in the aerosol concentration, which resulted in the measured curve. The RTD at the lower part of the reactor and in the simulation reacted to the step function with a delay, which can again be explained by the filling of the reactor with Luffa and the resulting slowing of the flow. For an idSTR and for the data collected in the upper part of the reactor no delay in the RTD could be observed. In this area of the reactor, the Luffa had no influence on the flow and therefore on the RTD.

The CDF function was similar for all experimental points and for the simulated data but differed more significantly from the CDF function of the idSTR. Therefore, the CDF function for a “real” STR was calculated according to Müller‐Erlwein (2015) by taking the dead space volume, which was determined from the experimental data, into account when calculating the CDF function. The CDF function determined in this way was similar to that determined experimentally and simulatively. The average residence time was determined using the trapezoidal rule. This resulted in 238.98 s in the simulation, 201.74 s in the experiment in the upper part, 215.29 s in the experiment in the lower part, and 287.42 s for an idSTR. The differences in the mean residence time can be explained, as in the calculation of the RTD function and the CDF, by dead spaces in the abPBR, which are not considered in the calculation for an idSTR. Depending on where the moisture was measured, there were also smaller differences in the mean residence time and the simulation also deviated from the experiments. Therefore, the actual reactor volume and thus also the proportion of dead spaces was calculated for all setups. These resulted in a dead volume of 46.89% for the simulation, 55.17% for the experiment in the upper part, and 52.16% for the experiment in the lower part. Differences between the simulation and the experiments mainly result from the fact that assumptions must be made for the simulation, such as the physical properties of the Luffa, which do not entirely correspond to the conditions in reality. To simulate the flow through the Luffa, the permeability had to be determined. For this purpose, a µCT scan of an exemplary piece of Luffa with an edge length of 1 × 1 cm was prepared (see Figure 4B) and used to theoretically determine the permeability. Due to the heterogeneity of the Luffa, this permeability is of course only an auxiliary value for the simulation and deviates from the actual values, which explains the differences between simulation and experiment. Furthermore, the experimental setup only allowed the determination of the tracer concentration at a single point in the reactor. In addition, the arrangement of the Luffa, whereby two Luffa never have the same dimensions or porosity, will also have an influence on the distribution of the aerosol and thus on the residence time. If there are gaps between the Luffa, short‐circuit flows can occur. The Luffa in the simulation were distributed evenly and average values were assumed for the physical properties. Furthermore, the coalescence of the aerosol particles and the evaporation were neglected in the simulation. Interactions between the aerosol particles were neglected as well and aerosol particles touching a surface were assumed to be absorbed in the liquid phase. The formation of liquid films can also be observed in experiments, although an exact description of the interaction of the aerosol with liquid phases was not possible. In the simulation, a steady flow was assumed and interactions with the aerosol particles were neglected. A vortex formation above the Luffa could be detected (see Figure 4D), which led to a good mixing of the aerosol and thus presumably improved the supply of aerosol to the biofilm. Dead spaces could be identified in the simulation mainly in the area between the upper connections and in the corners of the reactor. However, as no cultivation takes place in these areas, these can be neglected and there is no need for further optimization.

### 3.3 Performance Evaluation of the New abPBR

#### 3.3.1 Comparison With Established Biofilm PBRs

To evaluate the performance of new bioreactors, it is useful to compare them with similar, already established systems. As the reactor presented here is an aerosol‐based biofilm PBR, there are only a few comparable systems based on the same principle. Therefore, a comparison was also carried out with biofilm PBRs based on submerged cultivation of the biofilm. Care was taken to ensure that the same strain *Nostoc* sp. was cultivated in all systems (even when using literature data). The following reactors were used for comparison: (i) stirred tank reactor (Minifors, Infors HT, Bottmingen, Switzerland), (ii) lab‐scale moving bed PBR (lsMBPBR) (Walther et al. 2022), (iii) small pilot‐scale MBPBR (psMBPR) (Walther et al. 2022), and (iv) emersed PBR (ePBR) (Strieth et al. 2021). The growth surfaces used for the STR, the MBPBRs, and the bubble column were HXF14‐KLL carriers (Hel‐X Biocarriers, Christian Stöhr GmbH & Co. KG), which are made of HDPE. Three different materials were used as growth surfaces for the ePBR: glass, PMMA, and silicone. In the abPBR developed here, Luffa was used as the growth body, as described above. Except for the stirred tank, all reactor types were developed for the phototrophic cultivation of biofilms and are therefore ideally suited as a reference system for evaluating the performance of the newly developed abPBR.

The reference value to which the growth of the biofilm is related may have a major influence on the assessment of performance. As not all reactors have the same volume or the same growth area, a direct comparison of the biomass produced over time is not possible without a relation to a reference value. Here, the ATY in relation to the cultivation surface was used for evaluation, as this is a decisive factor in the assessment of biofilm reactors. Biofilm reactors should be designed so that most of the biomass grows on a certain surface area, which is why this growth area is a decisive parameter for biofilm PBR. As the space requirement of a reactor is important as well, the ATY was also determined in relation to the footprint of the reactor. The productivity in relation to the medium volume was used as a third comparative parameter.

**Figure 5:**
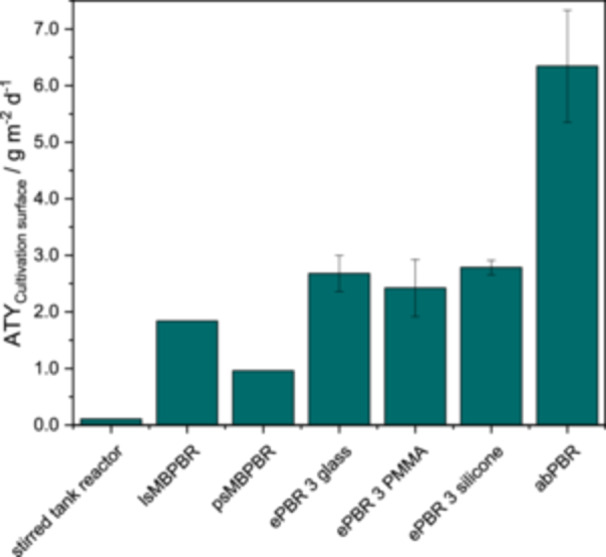
Area time yield based on the cultivation surface for the cultivation of *Nostoc* sp. in different biofilm‐based photobioreactors. Media supply via a submerged cultivation (stirred tank reactor, lab‐scale moving bed PBR (lsMBPBR) and pilot‐scale moving bed PBR (psMBPBR) (Walther et al. 2022) or via an aerosol produced with an ultrasonic transducer (emersed PBR [ePBR] with different cultivation surfaces [Strieth et al. 2021] and aerosol‐based PBR with Luffa [abPBR]). *n* = 3.

When looking at the ATY_cultivation surface_ (see Figure 5), it becomes clear that the growth of the strain *Nostoc* sp. in biofilm form in aerosol‐based reactors leaded to higher ATY_cultivation surface_ compared to submerged reactors. Since *Nostoc* sp. is a terrestrial strain, cultivation in the aerosol can better imitate the natural growth form, which can lead to better growth. When comparing the newly developed abPBR with other aerosol‐based systems (ePBR), an increase in ATY_cultivation surface_ of 2.3 times could be observed. As other substrates were used in the ePBR, this difference can of course also result from the good growth of cyanobacteria on Luffa (Kollmen et al. 2023). However, as the ePBR is designed for cultivation surfaces in the form of flat supports, cultivation on Luffa in the ePBR is not feasible for comparison. This shows that the overall concept of the abPBR for cultivation on Luffa is excellently suited for cultivation of terrestrial cyanobacteria. To calculate the ATY_cultivation surface_, a mean specific surface area of the Luffa of 9.50 cm^−2^ cm^−3^ was used as a basis. This is only an assumption based on literature data (In‐na et al. 2020). If, for example, the µCT scan (Figure 4B) of Luffa prepared in this study is considered, this results in a specific surface area of 16.71 cm^−2^ cm^−3^ assuming that the inner pores are filled. With this specific surface area, the ATY_cultivation surface_ is reduced to 3.60 g m^−2^ d^−1^, which is significantly smaller than the previously calculated 6.34 g m^−2^ d^−1^, but still larger than the ATY_cultivation surface_ in the other reactors. For further discussion, the average literature value will be used, as this is probably closer to an average value for Luffa as a larger section of the Luffa is considered here.

As previously mentioned, the evaluation of the performance of a reactor depends on the reference value in relation to which comparative values are determined. To obtain an evaluation of the performance of the abPBR that is as universal as possible, other comparative values were therefore also considered (see Table 1). This showed that the abPBR enables a more than 50% higher ATY in relation to the reactor footprint compared to previously developed abPBRs and MBPBRs. Only when considering the space‐time‐yield, no improvement could be achieved. With an STY of 0.114 g L^−1^ d^−1^, a slightly poorer STY was achieved compared to the ePBR (0.139 g L^−1^ d^−1^). It should be noted that the filling level of the abPBR was only approx. 50% in the test to ensure uniform aerosol distribution. An increase in the filling level would have a direct effect on the STY, which could be further optimized in future cultivations. However, an influence on the ATY in relation to the cultivation area cannot be ruled out due to poorer aerosol distribution. However, an advantage of the aerosol‐based reactors becomes clear in both systems. Due to the recycling of the medium and the fact that a large space with a small volume of medium can be filled with an aerosol, the required medium volume can be significantly reduced compared to submerged systems. As a result, the STY in the abPBR could be increased more than sixfold compared to the psMBPBR. In addition to the previously compared biofilm PBRs, Table 1 contains an overview of other biofilm PBRs described in the literature. Where possible, all three comparative values (ATY_cultivation surface_, ATY_footprint_ and STY) were calculated. In general, biofilm PBRs were listed, regardless of whether cyanobacteria or microalgae were cultivated in them and regardless of whether the biofilm was air‐exposed, cultivated in an aerosol‐based system, or submerged. Comparability is therefore limited, although the table allows a good placement of the abPBR in the context of existing systems. For example, the ATY_cultivation surface_, which is particularly important for biofilm reactors, shows that of the 31 reactors for which a calculation was possible based on the available data, only four reactors have a higher value compared to the abPBR. At 20.1 g m^−2^ d^−1^, the rotating biofilm contactor (RBC) according to Blanken et al. (2014) has the highest ATY of all reactors. It is based on rotating disks made of a stainless‐steel woven mesh, which partially extend into the medium and on which the biofilm grows. The experiments were carried out with *Chlorella sorokiniana*, a microalgae. During cultivation, a partial harvest was carried out after 7 days, which may be one of the reasons for the high ATY, as partial harvests can increase productivity (Boelee et al. 2014). In the abPBR, cultivation was carried out for 14 days without intermediate harvests. In general, a comparison with other biofilm PBRs shows that the abPBR is already performing at a high level in its current stage of development. It should be noted that no further optimization of the reactor has yet taken place. It would be conceivable to change the flow rate of the aerosol, the filling level, or the cultivation period. Partial harvests would also be imaginable and could increase yields. However, it is particularly important to adapt the parameters of light intensity and cultivation temperature to the respective strain, which is easy to implement due to the design of the reactor with external lighting and temperature control, which is why the abPBR should show good adaptability for cultivating other strains.

#### 3.3.2 Potential for Application‐Related Conditioning of Biofilms

Biofilms are exposed to many different stressors in nature and have developed tactics to survive despite external stress factors. In biotechnological cultivations, stress responses of the biofilm may not only be undesirable but may also contribute to an increased formation of the target product. For example, the production of secondary metabolites in plants can be increased by drought stress (Selmar and Kleinwächter 2013). This has not yet been extensively studied for cyanobacteria. However, other applications are conceivable, such as the production of increased EPS content through external stress and either the subsequent use of the EPS as a product or the use of the biofilm with increased EPS content. This could make the biofilm more robust, as the EPS take over important protective mechanisms in the biofilm. While physical stress factors such as heat stress or light stress are more or less easy to demonstrate in almost all PBRs, drought stress is difficult to realize in submerged systems, as this would require a separation of biomass and medium. This separation already exists in biofilm PBR and especially in aerosol‐based systems, where the medium is present in an external reservoir. Drought stress is therefore very easy to realize. All that is required here is to switch off the medium supply for a certain period of time. In this study, the influence of a rhythmically controlled aerosol supply in the abPBR was investigated with the cyanobacteria *Nostoc* sp. and *D. muscorum*. In the cultivation with *Nostoc* sp. the aerosol supply was switched on for 12 h and then switched off for 12 h. In the cultivation with *D. muscorum*, a rhythm of 4 h on and 4 h off was selected. In both cases, there was no aerosol for 50% of the time and consequently drought stress was present.

As expected, drought stress led to reduced growth of cyanobacteria (see Table 2). This result was independent of the strain used. For instance, in cultivation with *Nostoc* sp., the ATY decreased significantly by approx. 15% and in cultivation with *D. muscorum* even by approx. 28%. This result was not to be expected, as the drought stress should be lower when cultivating with *D. muscorum* due to the shorter on/off cycles. Therefore, a lower decrease in the ATY of *D. muscorum* would have been expected. The fact that this was not the case may indicate that *Nostoc* sp. is more tolerant to drought stress than *D. muscorum*. However, this must be proven by further experiments. The growth of the cyanobacteria already showed that all the effects determined here are strain‐specific and cannot easily be transferred to other strains. This becomes even clearer when the EPS content is considered. The EPS serve as a water reservoir for the cyanobacteria, which is why it would be expected that they would produce more of it under drought stress (Nishanth et al. 2021). This result was also achieved in the cultivation with *D. muscorum*. The EPS content in the cultivation with drought stress was 50% higher than in the cultivation without drought stress. However, the opposite results were obtained with *Nostoc* sp. Here the EPS content was reduced by more than 50% with drought stress. Lan et al. (2010) investigated the effect of drought stress on man‐made cyanobacterial crusts and looked at the EPS content in the biofilm. They observed that the EPS content in the biofilm increased at the beginning of cultivation under drought stress and remained constant, whereas in the control the EPS content increased during cultivation. Therefore, they also obtained a lower EPS content with drought stress at the end of the cultivation. Furthermore, in this study, the time of harvest must also be considered, as the harvest was carried out shortly after the end of the drought cycle when cultivating with *Nostoc* sp. It is therefore possible that the content would be different at a different harvest time, which should be investigated in further trials. However, it can already be seen that growth and EPS content can be influenced strain‐specifically by drought stress. The phycobiliproteins were further extracted from the biomass, whereby an increase in the PBP content could be achieved in both strains through drought stress. The extent of the increase was again strain‐specific. The greatest increase was achieved in the APC content of *Nostoc* sp., which was more than doubled. This shows that drought stress over short time periods can lead to an increase in PBP content in cyanobacteria. There is only a small number of publications on the effect of drought stress on PBP content in cyanobacteria. Simeunović et al. (2013) investigated the PBP content in different *Nostoc* and *Anabaena* strains after 10 years of storage under drought stress in the dark. Even these strains still contained PBPs, albeit only at low levels. Nevertheless, the study shows the enormous tolerance of cyanobacteria to drought periods, which is why significant effects on product compositions could also be investigated by extending the drying cycles in the abPBR. The results achieved here show that the reactor and the cultivation system are not only excellently suited for cultivating terrestrial cyanobacteria, but also for specifically influencing the growth or productivity of products by means of drought stress.

**Table 2:** Comparison of the cultivation of *Nostoc* sp. and *D. muscorum* in the abPBR. Both strains were once cultivated with a continuous aerosol supply and once with a periodically aerosol supply.

	Unit	*Nostoc* sp.		*D. muscorum*	
		Aerosol supply			
		Continuously	12 h on/12 h off	Continuously	4 h on/4 h off
ATY	${\text{g}}_{\text{CDW}}\;{\text{m}}^{-2}\;{\text{d}}^{-1}$	6.343 ± 0.990^a^	5.360 ± 0.901^b^	4.196 ± 1.991^b,c^	3.009 ± 0.549^c^
CDW per m²	$\text{g}\;{\text{m}}^{-2}$	88.806 ± 13.856^a^	75.043 ± 12.612^b^	58.748 ± 27.874^b,c^	42.130 ± 7.688^c^
EPS	$\text{g}\;{\text{g}}_{\text{CDW}}^{-1}$	0.528 ± 0.164^a^	0.250 ± 0.044^b^	0.055 ± 0.010^c^	0.083 ± 0.005^d^
CPC	$\text{mg}\;{\text{g}}_{\text{CDW}}^{-1}$	62.087 ± 15.875^a^	83.925 ± 14.396^b^	17.921 ± 4.560^c^	19.413 ± 7.923^c^
APC	$\text{mg}\;{\text{g}}_{\text{CDW}}^{-1}$	10.734 ± 5.125^a^	22.388 ± 3.201^b,c^	13.576 ± 4.046^a^	15.431 ± 6.526^a,c^
PE	$\text{mg}\;{\text{g}}_{\text{CDW}}^{-1}$	2.548 ± 0.697^a^	1.702 ± 2.069^a^	13.135 ± 3.153^b^	15.208 ± 4.649^b^

*Note:* The Mann‐Whitney *U* test was performed to determine whether area time yield (ATY) based on the cultivation surface, the cell dry weight (CDW) per m2 of Luffa surface, the EPS, C‐phycocyanin (CPC), allophycocyanin (APC) and phycoerythrin (PE) is statistically identical. H0: The difference in the position under the sample is zero. If the *p* value is higher than 0.05, the hypothesis can be discarded. Small letters (a–c) indicate significant differences (*p* < 0.05). *n* = 8.

The potential of abPBR has certainly not yet been exhausted and should not be limited to the cultivation of cyanobacteria. In‐na et al. (2020) used a biocomposite of living microalgae or cyanobacteria immobilized in a latex layer on Luffa for CO_2_ capture. The Luffa coated with the biofilm were placed in a glass with medium, which was absorbed by the Luffa and thus enabled the organisms to be supplied with medium. The glasses were sealed and gassed with CO_2_− enriched air. In‐na et al. (2020) were able to show that CO_2_ capture can be intensified by the porous structure of this living biocomposite. In a further study, In‐na et al. (2022) carried out a techno‐economic analysis of CO_2_ capture from breweries using the biocomposite. They compared their system with commercial systems in the form of an open raceway, a flat‐panel PBR and a biofilm PBR. The comparison systems were all illuminated with sunlight, while the biocomposites were illuminated with artificial light with a lower PAR. Nevertheless, the biocomposites performed comparably well to the best commercial system, the biofilm PBR, although CO_2_ capture was worse due to lower light availability. The savings result from a significant reduction in water and energy demand of the biocomposites. The reference system used is the Algae biofilm PBR according to Ozkan et al. (2012) which is also listed in Table 1. When comparing the ATY and STY of the reactor according to Ozkan et al. (2012) with the abPBR developed in this study, it becomes clear that both ATY and STY are approximately 10 times higher in the abPBR. The abPBR could be used without any restrictions for CO_2_ capture with the biocomposite according to In‐na et al. (2020), as the abPBR is designed for the use of commercially available Luffa sponges, which were also used to produce the biocomposites. Due to the outstanding ATY_cultivation surface_ compared to other biofilm PBRs (especially the Algae biofilm PBR by Ozkan et al. (2012), which was used as reference system by In‐na et al. [2022]), an application with biocomposites for CO_2_ capture could be promising. By optimizing the growth of cyanobacteria in the abPBR, CO_2_ capture can be increased and thus the economic efficiency will be improved. Of course, a techno‐economic analysis would have to be carried out for the specific case. Another possible application is the use for biosorption of heavy metals with microalgae or cyanobacteria immobilized on Luffa from wastewater, which has already been described in the literature (Akhtar et al. 2003a, 2004; Akhtar et al. 2008; Akhtar et al. 2003b). Here it would be conceivable to first cultivate the organisms on Luffa in the abPBR and then flood it with wastewater to carry out the biosorption in a batch process.

In conclusion, it can be stated that the abPBR developed in this study can be used for various applications. In addition to its excellent suitability for the immobilized cultivation of terrestrial cyanobacteria, the aerosol‐based reactor concept allows a simple simulation of drought stress. Immobilization on Luffa can also be used as a promising concept for biosorption, for example, or biocomposites can be produced from cyanobacteria and microalgae with Luffa for CO_2_ capture. Further applications are conceivable and are left to the reader's own creativity.

## 4 Conclusion

In this study, a novel abPBR for cultivating cyanobacteria on biodegradable Luffa was successfully developed and characterized. By leveraging the unique properties of Luffa as a growth substrate, the reactor design ensured effective light, humidity, and temperature distribution, enabling optimal growth conditions for the biofilms. The RTD was determined both experimentally and simulatively, whereby the mean residence times are similar. The supply of medium in form of an aerosol not only supported the growth of terrestrial cyanobacteria such as *Nostoc* sp. and *D. muscorum* but also provided a flexible platform to simulate environmental stress conditions, such as drought, in a controlled manner.

The reactor demonstrated high productivity, achieving maximum ATYs of 6.34 and 4.19 g m^−^² d^−^¹ for *Nostoc* sp. and *D. muscorum*, respectively. Comparative performance analysis revealed that the abPBR outperforms many established biofilm PBR systems in terms of ATY, based on both the cultivation surface and the footprint of the reactor. Additionally, the ability to induce and study stress responses, such as drought‐induced variations in EPS and phycobiliprotein production, highlights the potential of the abPBR for application‐oriented biofilm conditioning.

Future optimization of the reactor, including adjustments to aerosol flow rates, filling levels, and cultivation cycles, could further enhance productivity and adaptability for different strains. The integration of a procedure for partial harvesting of the biofilm, for example, by washing off the biomass, could lead to an increase in productivity. The application possibilities of abPBR are not limited to cultivation but can be extended to CO_2_ capture with cyanobacteria Luffa biocomposites, for example. This study underscores the promise of the “Luffa abPBR” as a sustainable and versatile platform for both fundamental research and industrial applications in biotechnology. The upscaling of the abPBR represents the main challenges and limitations for future applications.

## Author Contributions

**Jonas Kollmen:** supervision, validation, writing – original draft, conceptualization, visualization, methodology, investigation, data curation. **Andreas Stiefelmaier:** software, data curation, visualization. **Ramtin Mofrad:** investigation. **Dorina Strieth:** funding acquisition, project administration, writing – review and editing, supervision, conceptualization.

## Data Availability

The authors declare that the data supporting the findings of this study are available within the paper. Should any raw data files be needed in another format they are available from the corresponding author upon reasonable request.

## References

[bit28992-bib-0001] Ahmad, I. , N. Abdullah , I. Koji , A. Yuzir , and S. Eva Muhammad . 2021. “Evolution of Photobioreactors: A Review Based on Microalgal Perspective.” IOP Conference Series: Materials Science and Engineering 1142, no. 1: 012004. 10.1088/1757-899X/1142/1/012004.

[bit28992-bib-0002] Akhtar, N. , J. Iqbal , and M. Iqbal . 2003a. “Microalgal‐Luffa Sponge Immobilized Disc: A New Efficient Biosorbent for the Removal of Ni(II) From Aqueous Solution.” Letters in Applied Microbiology 37, no. 2: 149–153. 10.1046/j.1472-765X.2003.01366.x. 12859658

[bit28992-bib-0003] Akhtar, N. , J. Iqbal , and M. Iqbal . 2004. “Enhancement of Lead(II) Biosorption by Microalgal Biomass Immobilized Onto Loofa (*Luffa cylindrica*) Sponge.” Engineering in Life Sciences 4, no. 2: 171–178. 10.1002/elsc.200420019.

[bit28992-bib-0004] Akhtar, N. , M. Iqbal , S. I. Zafar , and J. Iqbal . 2008. “Biosorption Characteristics of Unicellular Green Alga *Chlorella sorokiniana* Immobilized in Loofa Sponge for Removal of Cr(III).” Journal of Environmental Sciences 20, no. 2: 231–239. 10.1016/S1001-0742(08)60036-4. 18574966

[bit28992-bib-0005] Akhtar, N. , A. Saeed , and M. Iqbal . 2003b. “ *Chlorella sorokiniana* Immobilized on the Biomatrix of Vegetable Sponge of Luffa Cylindrica: A New System to Remove Cadmium From Contaminated Aqueous Medium.” Bioresource Technology 88, no. 2: 163–165. 10.1016/S0960-8524(02)00289-4. 12576011

[bit28992-bib-0006] Alhijazi, M. , B. Safaei , Q. Zeeshan , M. Asmael , A. Eyvazian , and Z. Qin . 2020. “Recent Developments in Luffa Natural Fiber Composites: Review.” Sustainability 12, no. 18: 7683. 10.3390/su12187683.

[bit28992-bib-0007] de Assis, L. R. , M. L. Calijuri , P. P. Assemany , E. C. Berg , L. V. Febroni , and T. A. Bartolomeu . 2019. “Evaluation of the Performance of Different Materials to Support the Attached Growth of Algal Biomass.” Algal Research 39: 101440. 10.1016/j.algal.2019.101440.

[bit28992-bib-0008] Blanken, W. , M. Janssen , M. Cuaresma , Z. Libor , T. Bhaiji , and R. H. Wijffels . 2014. “Biofilm Growth of *Chlorella sorokiniana* in a Rotating Biological Contactor Based Photobioreactor.” Biotechnology and Bioengineering 111, no. 12: 2436–2445. 10.1002/bit.25301. 24895246

[bit28992-bib-0009] Boelee, N. C. , M. Janssen , H. Temmink , et al. 2014. “The Effect of Harvesting on Biomass Production and Nutrient Removal in Phototrophic Biofilm Reactors for Effluent Polishing.” Journal of Applied Phycology 26, no. 3: 1439–1452. 10.1007/s10811-013-0178-1. 24081706

[bit28992-bib-0010] Chang, J. ‑S. , P. ‑L. Show , T. ‑C. Ling , et al. 2017. “Photobioreactors.” In Current Developments in Biotechnology and Bioengineering, 313–352. Elsevier. 10.1016/B978-0-444-63663-8.00011-2.

[bit28992-bib-0011] Cheng, P. , B. Ji , L. Gao , W. Zhang , J. Wang , and T. Liu . 2013. “The Growth, Lipid and Hydrocarbon Production of *Botryococcus braunii* With Attached Cultivation.” Bioresource Technology 138: 95–100. 10.1016/j.biortech.2013.03.150. 23612166

[bit28992-bib-0012] Cheng, P. , D. Osei‐Wusu , C. Zhou , et al. 2020. “The Effects of Refractory Pollutants in Swine Wastewater on the Growth of Scenedesmus sp. With Biofilm Attached Culture.” International Journal of Phytoremediation 22, no. 3: 241–250. 10.1080/15226514.2019.1658706. 31475567

[bit28992-bib-0013] Choudhary, P. , S. K. Prajapati , P. Kumar , A. Malik , and K. K. Pant . 2017. “Development and Performance Evaluation of an Algal Biofilm Reactor for Treatment of Multiple Wastewaters and Characterization of Biomass for Diverse Applications.” Bioresource Technology 224: 276–284. 10.1016/j.biortech.2016.10.078. 27818159

[bit28992-bib-0014] Christenson, L. B. , and R. C. Sims . 2012. “Rotating Algal Biofilm Reactor and Spool Harvester for Wastewater Treatment With Biofuels by‐Products.” Biotechnology and Bioengineering 109, no. 7: 1674–1684. 10.1002/bit.24451. 22328283

[bit28992-bib-0015] Genin, S. N. , J. Stewart Aitchison , and D. Grant Allen . 2014. “Design of Algal Film Photobioreactors: Material Surface Energy Effects on Algal Film Productivity, Colonization and Lipid Content.” Bioresource Technology 155: 136–143. 10.1016/j.biortech.2013.12.060. 24441594

[bit28992-bib-0016] Gross, M. , and Z. Wen . 2014. “Yearlong Evaluation of Performance and Durability of a Pilot‐Scale Revolving Algal Biofilm (RAB) Cultivation System.” Bioresource Technology 171: 50–58. 10.1016/j.biortech.2014.08.052. 25189508

[bit28992-bib-0017] Gupta, P. L. , S. M. Lee , and H. J. Choi . 2015. “A Mini Review: Photobioreactors for Large Scale Algal Cultivation.” World Journal of Microbiology and Biotechnology 31, no. 9: 1409–1417. 10.1007/s11274-015-1892-4. 26085485

[bit28992-bib-0018] Hedges, S. B. , H. Chen , S. Kumar , D. Y. Wang , A. S. Thompson , and H. Watanabe . 2001. “A Genomic Timescale for the Origin of Eukaryotes.” BMC Evolutionary Biology 1, no. 1: 4. 10.1186/1471-2148-1-4. 11580860 PMC56995

[bit28992-bib-0019] Hornung, M. , M. Ludwig , and H. P. Schmauder . 2007. “Optimizing the Production of Bacterial Cellulose in Surface Culture: A Novel Aerosol Bioreactor Working on a Fed Batch Principle (Part 3).” Engineering in Life Sciences 7, no. 1: 35–41. 10.1002/elsc.200620164.

[bit28992-bib-0020] Iman Shayan, S. , F. A. Agblevor , L. Bertin , and R. C. Sims . 2016. “Hydraulic Retention Time Effects on Wastewater Nutrient Removal and Bioproduct Production via Rotating Algal Biofilm Reactor.” Bioresource Technology 211: 527–533. 10.1016/j.biortech.2016.03.104. 27038261

[bit28992-bib-0021] In‐na, P. , F. Byrne , G. S. Caldwell , and J. G. M. Lee . 2022. “Techno‐Economic Analysis of Living Biocomposites for Carbon Capture From Breweries.” Algal Research 66: 102781. 10.1016/j.algal.2022.102781.

[bit28992-bib-0022] In‐na, P. , A. A. Umar , A. D. Wallace , M. C. Flickinger , G. S. Caldwell , and J. G. M. Lee . 2020. “Loofah‐Based Microalgae and Cyanobacteria Biocomposites for Intensifying Carbon Dioxide Capture.” Journal of CO2 Utilization 42: 101348. 10.1016/j.jcou.2020.101348.

[bit28992-bib-0023] Ji, B. , W. Zhang , N. Zhang , J. Wang , G. A. Lutzu , and T. Liu . 2014. “Biofilm Cultivation of the Oleaginous Microalgae Pseudochlorococcum sp.” Bioprocess and Biosystems Engineering 37, no. 7: 1369–1375. 10.1007/s00449-013-1109-x. 24362561

[bit28992-bib-0024] Johnson, M. B. , and Z. Wen . 2010. “Development of an Attached Microalgal Growth System for Biofuel Production.” Applied Microbiology and Biotechnology 85, no. 3: 525–534. 10.1007/s00253-009-2133-2. 19636552

[bit28992-bib-0025] Kollmen, J. , M. Rech , F. Lorig , S. Di Nonno , J. Stiefelmaier , and D. Strieth . 2025. “New Easy Lab Methods for the Extraction of Phycobiliproteins and Pigments From Cyanobacteria.” Journal of Applied Phycology. 10.1007/s10811-024-03414-8.

[bit28992-bib-0026] Kollmen, J. , J. Stiefelmaier , R. Mofrad , and D. Strieth . 2023. “Cultivation of Cyanobacteria on Sustainable Dried Luffa Cylindrica.” Phycology 3, no. 4: 472–483. 10.3390/phycology3040032.

[bit28992-bib-0027] Kollmen, J. , and D. Strieth . 2022. “The Beneficial Effects of Cyanobacterial Co‐Culture on Plant Growth.” Life 12, no. 2: 223. 10.3390/life12020223. 35207509 PMC8879750

[bit28992-bib-0028] Kuhne, S. , D. Strieth , A. Weber , K. Muffler , M. Lakatos , and R. Ulber . 2014. “Screening of Two Terrestrial Cyanobacteria for Biotechnological Production Processes in Shaking Flasks, Bubble Columns, and Stirred Tank Reactors.” Journal of Applied Phycology 26, no. 4: 1639–1648. 10.1007/s10811-013-0224-z.

[bit28992-bib-0029] Lan, S. , L. Wu , D. Zhang , C. Hu , and Y. Liu . 2010. “Effects of Drought and Salt Stresses on Man‐Made Cyanobacterial Crusts.” European Journal of Soil Biology 46, no. 6: 381–386. 10.1016/j.ejsobi.2010.08.002.

[bit28992-bib-0030] Lee, S. H. , H. M. Oh , B. H. Jo , et al. 2014. “Higher Biomass Productivity of Microalgae in an Attached Growth System, Using Wastewater.” Journal of Microbiology and Biotechnology 24, no. 11: 1566–1573. 10.4014/jmb.1406.06057. 25112320

[bit28992-bib-0031] Li, W. , H. N. Su , Y. Pu , et al. 2019. “Phycobiliproteins: Molecular Structure, Production, Applications, and Prospects.” Biotechnology Advances 37, no. 2: 340–353. 10.1016/j.biotechadv.2019.01.008. 30685481

[bit28992-bib-0032] Lin‐Lan, Z. , W. Jing‐Han , and H. Hong‐Ying . 2018. “Differences Between Attached and Suspended Microalgal Cells in ssPBR From the Perspective of Physiological Properties.” Journal of Photochemistry and Photobiology, B: Biology 181: 164–169. 10.1016/j.jphotobiol.2018.03.014. 29571071

[bit28992-bib-0033] Melo, M. , S. Fernandes , N. Caetano , and M. T. Borges . 2018. “ *Chlorella vulgaris* (SAG 211‐12) Biofilm Formation Capacity and Proposal of a Rotating Flat Plate Photobioreactor for More Sustainable Biomass Production.” Journal of Applied Phycology 30, no. 2: 887–899. 10.1007/s10811-017-1290-4.

[bit28992-bib-0034] Mulbry, W. W. , and A. C. Wilkie . 2001. “Growth of Benthic Freshwater Algae on Dairy Manures.” Journal of Applied Phycology 13, no. 4: 301–306. 10.1023/A:1017545116317.

[bit28992-bib-0035] Müller‐Erlwein, E. 2015. “Verweilzeitverhalten.” In Chemische Reaktionstechnikedited by E. Müller‐Erlwein , 94–138. Springer Fachmedien Wiesbaden. 10.1007/978-3-658-09396-9_6.

[bit28992-bib-0036] Naumann, T. , Z. Çebi , B. Podola , and M. Melkonian . 2013. “Growing Microalgae as Aquaculture Feeds on Twin‐Layers: A Novel Solid‐State Photobioreactor.” Journal of Applied Phycology 25, no. 5: 1413–1420. 10.1007/s10811-012-9962-6.

[bit28992-bib-0037] Nishanth, S. , A. Bharti , H. Gupta , K. Gupta , U. Gulia , and R. Prasanna . 2021. “Cyanobacterial Extracellular Polymeric Substances (EPS): Biosynthesis and Their Potential Applications.” In Microbial and Natural Macromolecules, 349–369. Elsevier. 10.1016/B978-0-12-820084-1.00015-6.

[bit28992-bib-0038] Ogbonna, J. C. , Y. C. Liu , Y. K. Liu , and H. Tanaka . 1994. “Loofa (*Luffacylindrica*) Sponge as a Carrier for Microbial Cell Immobilization.” Journal of Fermentation and Bioengineering 78, no. 6: 437–442. 10.1016/0922-338X(94)90043-4.

[bit28992-bib-0039] Orandi, S. , D. M. Lewis , and N. R. Moheimani . 2012. “Biofilm Establishment and Heavy Metal Removal Capacity of an Indigenous Mining Algal‐Microbial Consortium in a Photo‐Rotating Biological Contactor.” Journal of Industrial Microbiology & Biotechnology 39, no. 9: 1321–1331. 10.1007/s10295-012-1142-9. 22644382

[bit28992-bib-0040] Ozkan, A. , K. Kinney , L. Katz , and H. Berberoglu . 2012. “Reduction of Water and Energy Requirement of Algae Cultivation Using an Algae Biofilm Photobioreactor.” Bioresource Technology 114: 542–548. 10.1016/j.biortech.2012.03.055. 22503193

[bit28992-bib-0041] Peng, Y. Y. , F. Gao , H. L. Yang , et al. 2020. “Simultaneous Removal of Nutrient and Sulfonamides From Marine Aquaculture Wastewater by Concentrated and Attached Cultivation of *Chlorella vulgaris* in an Algal Biofilm Membrane Photobioreactor (BF‐MPBR).” Science of the Total Environment 725: 138524. 10.1016/j.scitotenv.2020.138524. 32302854

[bit28992-bib-0042] Roselet, F. , P. Maicá , T. Martins , and P. C. Abreu . 2013. “Comparison of Open‐Air and Semi‐Enclosed Cultivation System for Massive Microalgae Production in Sub‐Tropical and Temperate Latitudes.” Biomass and Bioenergy 59: 418–424. 10.1016/j.biombioe.2013.09.014.

[bit28992-bib-0043] Rossi, F. , and R. De Philippis . 2015. “Role of Cyanobacterial Exopolysaccharides in Phototrophic Biofilms and in Complex Microbial Mats.” Life 5, no. 2: 1218–1238. 10.3390/life5021218. 25837843 PMC4500136

[bit28992-bib-0044] Scherer, K. , J. Stiefelmaier , D. Strieth , M. Wahl , and R. Ulber . 2020. “Development of a Lightweight Multi‐Skin Sheet Photobioreactor for Future Cultivation of Phototrophic Biofilms on Facades.” Journal of Biotechnology 320: 28–35. 10.1016/j.jbiotec.2020.06.004. 32533991

[bit28992-bib-0045] Schmidt, T. , M.‑K. Nguyen , and M. Lakatos . 2017. “Phototrophe Mikroorganismen an der Fassade.” Fassade 1: 24–26.

[bit28992-bib-0046] Schnurr, P. J. , G. S. Espie , and D. G. Allen . 2013. “Algae Biofilm Growth and the Potential to Stimulate Lipid Accumulation Through Nutrient Starvation.” Bioresource Technology 136: 337–344. 10.1016/j.biortech.2013.03.036. 23567700

[bit28992-bib-0047] Schwarz, A. , J. Walther , D. Geib , et al. 2020. “Influence of Heterotrophic and Mixotrophic Cultivation on Growth Behaviour of Terrestrial Cyanobacteria.” Algal Research 52: 102125. 10.1016/j.algal.2020.102125.

[bit28992-bib-0048] Selmar, D. , and M. Kleinwächter . 2013. “Stress Enhances the Synthesis of Secondary Plant Products: The Impact of Stress‐Related Over‐Reduction on the Accumulation of Natural Products.” Plant & Cell Physiology 54, no. 6: 817–826. 10.1093/pcp/pct054. 23612932

[bit28992-bib-0049] Shen, Y. , T. Yu , Y. Xie , et al. 2019. “Attached Culture of Chlamydomonas sp. JSC4 for Biofilm Production and TN/TP/Cu(II) Removal.” Biochemical Engineering Journal 141: 1–9. 10.1016/j.bej.2018.09.017.

[bit28992-bib-0050] Simeunović, J. , K. Bešlin , Z. Svirčev , D. Kovač , and O. Babić . 2013. “Impact of Nitrogen and Drought on Phycobiliprotein Content in Terrestrial Cyanobacterial Strains.” Journal of Applied Phycology 25, no. 2: 597–607. 10.1007/s10811-012-9894-1.

[bit28992-bib-0051] Singh, R. N. , and S. Sharma . 2012. “Development of Suitable Photobioreactor for Algae Production—A Review.” Renewable and Sustainable Energy Reviews 16, no. 4: 2347–2353. 10.1016/j.rser.2012.01.026.

[bit28992-bib-0052] Stal, L. J. 2005. “Nitrogen Fixation in Cyanobacteria.” In Encyclopedia of Life Sciences, 1–9. Wiley. 10.1002/9780470015902.a0021159.pub2.

[bit28992-bib-0053] Stanier, R. Y. , J. Deruelles , R. Rippka , M. Herdman , and J. B. Waterbury . 1979. “Generic Assignments, Strain Histories and Properties of Pure Cultures of Cyanobacteria.” Microbiology 111, no. 1: 1–61. 10.1099/00221287-111-1-1.

[bit28992-bib-0054] Stiefelmaier, J. 2020. Phototrophe Biofilme als Produktionssysteme (1st ed). Dr. Hut.

[bit28992-bib-0055] Strieth, D. , A. Weber , J. Robert , et al. 2021. “Characterization of an Aerosol‐Based Photobioreactor for Cultivation of Phototrophic Biofilms.” Life 11, no. 10: 1046. 10.3390/life11101046. 34685417 PMC8538940

[bit28992-bib-0056] Strieth, D. 2019. *Produktive phototrophe Biofilme in Aerosolreaktoren* (1. Auflage). Verfahrenstechnik. Verlag Dr. Hut.

[bit28992-bib-0057] Sukačová, K. , M. Trtílek , and T. Rataj . 2015. “Phosphorus Removal Using a Microalgal Biofilm in a New Biofilm Photobioreactor for Tertiary Wastewater Treatment.” Water Research 71: 55–63. 10.1016/j.watres.2014.12.049. 25594825

[bit28992-bib-0058] Tao, Q. , F. Gao , C. Y. Qian , X. Z. Guo , Z. Zheng , and Z. H. Yang . 2017. “Enhanced Biomass/Biofuel Production and Nutrient Removal in an Algal Biofilm Airlift Photobioreactor.” Algal Research 21: 9–15. 10.1016/j.algal.2016.11.004.

[bit28992-bib-0059] Tscheschke, B. , J. Dreimann , J. W. von der Ruhr , et al. 2015. “Evaluation of a New Mist‐Chamber Bioreactor for Biotechnological Applications.” Biotechnology and Bioengineering 112, no. 6: 1155–1164. 10.1002/bit.25523. 25545471

[bit28992-bib-0060] de Vree, J. H. , R. Bosma , M. Janssen , M. J. Barbosa , and R. H. Wijffels . 2015. “Comparison of Four Outdoor Pilot‐Scale Photobioreactors.” Biotechnology for Biofuels 8: 215. 10.1186/s13068-015-0400-2. 26689675 PMC4683866

[bit28992-bib-0061] Walther, J. , N. Erdmann , M. Stoffel , et al. 2022. “Passively Immobilized Cyanobacteria Nostoc Species BB 92.2 in a Moving Bed Photobioreactor (MBPBR): Design, Cultivation and Characterization.” Biotechnology and Bioengineering 119: 1467–1482. 10.1002/bit.28072. 35211957

[bit28992-bib-0062] Weathers, P. , C. Liu , M. Towler , and B. Wyslouzil . 2008. “Mist Reactors: Principles, Comparison of Various Systems, and Case Studies.” Electron J Integr Biosci 3, no. 1: 29–37.

[bit28992-bib-0063] Xu, H. , H. Cai , G. Yu , and H. Jiang . 2013. “Insights Into Extracellular Polymeric Substances of Cyanobacterium *Microcystis aeruginosa* Using Fractionation Procedure and Parallel Factor Analysis.” Water Research 47, no. 6: 2005–2014. 10.1016/j.watres.2013.01.019. 23395483

[bit28992-bib-0064] Xu, X. Q. , J. H. Wang , T. Y. Zhang , G. H. Dao , G. X. Wu , and H. Y. Hu . 2017. “Attached Microalgae Cultivation and Nutrients Removal in a Novel Capillary‐Driven Photo‐Biofilm Reactor.” Algal Research 27: 198–205. 10.1016/j.algal.2017.08.028.

[bit28992-bib-0065] Yin, S. , J. Wang , L. Chen , and T. Liu . 2015. “The Water Footprint of Biofilm Cultivation of *Haematococcus pluvialis* Is Greatly Decreased by Using Sealed Narrow Chambers Combined With Slow Aeration Rate.” Biotechnology Letters 37, no. 9: 1819–1827. 10.1007/s10529-015-1864-7. 25994585

[bit28992-bib-0066] Zamalloa, C. , N. Boon , and W. Verstraete . 2013. “Decentralized Two‐Stage Sewage Treatment by Chemical‐Biological Flocculation Combined With Microalgae Biofilm for Nutrient Immobilization in a Roof Installed Parallel Plate Reactor.” Bioresource Technology 130: 152–160. 10.1016/j.biortech.2012.11.128. 23306123

